# Hereditary connective tissue disorders in unselected patients with spontaneous cervical artery dissection: a targeted next generation sequencing approach and systematic review

**DOI:** 10.1007/s10072-026-09308-6

**Published:** 2026-09-02

**Authors:** Lorenzo Corradi, Chiara Ferraro, Fiammetta Tesi, Giorgia Abrignani, Paola Castellini, Lilia Latte, Maria Claudia Trapasso, Antonio Genovese, Marco Giuseppe Ritelli, Valeria Cinquina, Silvia Clara Giliani, Mauro Magoni, Roberto Menozzi, Alessandro Pezzini

**Affiliations:** 1 Department of Medicine and Surgery, University of Parma, Parma, Italy; 2 Stroke Care Program, Department of Emergency, Parma University Hospital, Parma, Italy; 3 Department of Molecular and Translational Medicine, Medical Genetics, University of Brescia, Brescia, Italy; 4 Department of Molecular and Translational Medicine, Nocivelli Institute for Molecular Medicine, University of Brescia, Brescia, Italy; 5 Department of Neurological Sciences and Vision, Vacular Neurology, ASST Spedali Civili, Brescia, Italy; 6 Diagnostics Department, Neuroradiology Unit, Parma University Hospital, Parma, Italy

**Keywords:** Dissection, arterial, Hereditary connective tissue disorders, Extracellular matrix, Stroke, ischemic, Next generation sequencing

## Abstract

**Introduction:**

Whether spontaneous cervical artery dissection (sCeAD), the leading cause of ischemic stroke in young adults, represents the manifestation of unrecognized hereditary connective tissue disorders (HCTDs) and whether HCTDs have a major impact in the epidemiology of the disease is a matter of ongoing debate. We aimed at determining the frequency of clinically relevant genetic variants (CRGVs) in a cohort of unselected sCeAD patients by targeted next-generation sequencing (NGS) approach.

**Methods:**

We designed a high-throughput sequencing panel to identify variants in 38 candidate genes associated with arterial dissection or aneurysm and screened patients with apparently sporadic sCeAD, consecutively referred to one comprehensive stroke center from August 2020 to December 2025. The frequency of known disease-causing and pertinent variants of uncertain significance (VUS) was calculated. Then, we performed a systematic review of all studies evaluating the prevalence of monogenic disorders among sCeAD patients up to December 2025.

**Results:**

Among 183 patients (males, 51.3%; mean age, 42.0 ± 11.3 years), 2 (1.1%) carried a CeAD-causing variant in COL3A1 (NM_000090.4:c.2959G > A:p.Gly987Ser) and ABCC6 (NM_001351800.1:c.3071G > A:p.Arg1024Gln), respectively. In addition, we identified 30 (16.4%) VUS in 25 (13.6%) patients. The analysis of 330 patients across 14 studies yielded a prevalence of carriers of disease-causing variants ranging between 0.4% in series of unselected patients and 23.4% in patients with familial history of CeAD.

**Conclusion:**

Systematic search for rare disease-causing variants should not be recommended in all sCeAD cases but it should be limited to selected individuals with a high pre-test probability to harbor a monogenic disease.

**Supplementary Information:**

The online version contains supplementary material available at https://doi.org/10.1007/s10072-026-09308-6.

## Introduction

Arterial dissection is, by definition, the accumulation of blood within the wall of an artery. Once considered uncommon, dissections of carotid or vertebral arteries (cervical artery dissections, CeADs) are now recognized as the major cause of ischemic stroke in young adults, accounting for approximately 20% of cases in patients under 45 years of age. Notwithstanding, the pathogenesis of CeAD is still poorly defined, especially in those cases that occur spontaneously (spontaneous CeAD, sCeAD), without any identifiable precipitating events [1]. Several arguments point toward a key role played by the connective tissue component of the arterial wall. The finding of composite collagen fibrils and fragmented elastic fibers on electron microscopic examination of skin biopsy specimens in more than half of patients with sCeAD [2–4] and the identification of clinically detectable signs of connective tissue aberration in most sCeAD patients support the hypothesis of a generalized structural connective tissue defect predisposing to disease occurrence, even in sporadic cases without evident signs of a known hereditary connective tissue disorder (HCTD) [5, 6]. In this view, the reduced or altered biosynthesis of the extracellular matrix (ECM) components might lead to a generalized structural weakness of the vessel wall and explain the increased risk for sCeAD. Whether such arteriopathy underlying sCeAD represents a mild phenotypic manifestation of HCTD has never been fully elucidated. Most of the studies so far have been conducted on small series with limited number of screened genes [7, 8]. Recently, next-generation sequencing (NGS) has become an effective tool to detect rare monogenic disorders. However, only a few NGS studies targeting series of sCeAD patients have been conducted thus far [9, 10]. Furthermore, such analyses were performed on highly selected groups of patients, based on family history of arterial dissection or morphological abnormalities in dermal connective tissue and are, therefore, unlikely to provide reliable information on the majority of sCeAD cases. Based on these premises, we aimed at investigating the prevalence of monogenic disorders in an unselected series of patients with sCeAD by a targeted NGS approach.

## Materials and methods

The recruitment period for the present study was August 2020 through December 2025. The study was approved by the ethics committee of Brescia University Hospital and all aspects were conducted in accordance with the Declaration of Helsinki. Written informed consent was obtained from all participants (or legal guardian) prior to enrollment. All consecutive patients who received a diagnosis of sCeAD were considered potentially eligible for the present study.

### Diagnosis of CeAD

Four-vessel conventional angiography, MRI with magnetic resonance angiography (MRA; 3-dimensional time of flight) and/or CT angiography of the brain and neck were included in the diagnostic workup. The presence of the double-lumen sign (a false lumen or an intimal flap), luminal narrowing with the “string sign,” and gradual tapering ending in total occlusion of the lumen (flame-like occlusion) were considered reliable angiographic findings of CeAD, whereas a narrowed lumen surrounded by a semilunar-shaped intramural hematoma on axial T1-weighted images was considered the pathognomonic MR sign [11].

### Definition of spontaneous CeAD

History of traumatic events before CeAD occurrence was collected in all patients. We considered mechanisms of trauma associated with CeAD: (1) any direct mechanical impact to the neck region; or (2) any impact to the head with indirect involvement of the neck region; or (3) any mechanical activity causing extraordinarily increased intra-thoracic pressure (e.g., heavy lifting), which had occurred within 1 month prior to first symptoms of dissection. Traumatic events leading to medical examination or hospitalization were considered “major” and all others were “minor” [11]. Dissections occurring as an immediate consequence of a major trauma were excluded.

### Construction of the panel

A targeted NGS panel approach was applied to all subjects who qualified for the analysis. We analyzed variants in 38 candidate genes known to be associated with arterial dissection (any site) or aneurysm, according to the Online Mendelian Inheritance of Men (OMIM) database (Table 1). Pathogenic variants in these genes caused monogenic disorders associated with arterial phenotypes including Ehlers–Danlos syndrome (EDS), Marfan syndrome, Loeys-Dietz syndrome (LDS), Alport syndrome, familial thoracic aortic aneurysms and dissections (TAAD), and arterial tortuosity syndrome (ATS). Blood samples were collected from all participants and DNA was extracted from ethylenediaminetetraacetic acid samples. After preparation of Ampliseq on demand amplicons, the NGS panel was run using IonChef automatic libraries preparation on a Ion GeneStudio™ S5 Prime (ThermoFisher Scientific). With vertical coverage under 30 reads, samples have been Sanger sequenced for the specific exons. Every variation found by NGS was confirmed by Sanger sequencing [12]. The detected variants were reclassified based on the interpretation guidelines from the American College of Medical Genetics and Genomics (ACMG) [13] into clinically relevant genetic variants (CRGVs; pathogenic or likely pathogenic) and VUS.

**Table 1:** Genes associated with arterial aneurysm or dissection included in the next-generation sequencing panel

Gene	Location	OMIM ID	Inheritance	Associated Mendelian Disorder
ABCC6	16p13.11	603,234	AR; AD	Arterial calcification, generalized, of infancy, 2; Pseudoxanthoma elasticum
ACTA2	10q23.31	102,620	AD	Moyamoya disease, Aortic aneurysm, familial thoracic; Multisystemic smooth muscle dysfunction syndrome
ADAR	1q21.3	146,920	AR	Dyschromatosis symmetrica hereditaria; Aicardi-Goutieres syndrome 6
COL3A1	2q32.2	120,180	AD; AR	Ehlers-Danlos syndrome, vascular type; Polymicrogyria with or without vascular-type Ehlers-Danlos syndrome
COL4A1	13q34	120,130	AD	Angiopathy, hereditary, with nephropathy, aneurysms, and muscle cramps; Brain small vessel disease with or without ocular anomalies; Anterior segment dysgenesis with cerebral involvement Porencephaly 1; Retinal artery tortuosity
COL4A2	13q34	120,090	AD	Brain small vessel disease 2 A (AD) and 2B (AR)
COL4A3	2q36.3	120,070	AD; AR	Alport syndrome 3 A, autosomal dominant; Alport syndrome 3B, autosomal recessive; Hematuria, benign familial, 2
COL4A4	2q36.3	120,131	AR; AD	Alport syndrome 2, autosomal recessive; Hematuria, familial benign, 1
COL4A5	Xq22.3	303,630	XLD	Alport syndrome 1, X-linked
COL5A1	9q34.3	120,215	AD	Ehlers-Danlos syndrome, classic type, 1; Fibromuscular dysplasia, multifocal
COL5A2	2q32.2	120,190	AD	Ehlers-Danlos syndrome, classic type, 2
EFEMP2	11q13.1	604,633	AR	Cutis laxa, autosomal recessive, type IB
ELN	7q11.23	130,160	AD	Cutis laxa, autosomal dominant; Supravalvar aortic stenosis
FBLN5	14q32.12	604,580	AD; AR	Cutis laxa, autosomal dominant 2; Charcot-Marie-Tooth disease, demyelinating, type 1 H; Cutis laxa, autosomal recessive, type IA; Macular degeneration, age-related, 3
FBN1	15q21.1	134,797	AD	Acromicric dysplasia; Ectopia lentis, familial; Geleophysic dysplasia 2; Marfan lipodystrophy syndrome; Marfan syndrome; MASS syndrome; Stiff skin syndrome; Weill-Marchesani syndrome 2, dominant
FLNA	Xq28	300,017	XL; XLR; XLD	FG syndrome 2; Cardiac valvular dysplasia, X-linked; Congenital short bowel syndrome; Frontometaphyseal dysplasia 1; Heterotopia, periventricular, 1; Intestinal pseudoobstruction, neuronal; Melnick-Needles syndrome; Otopalatodigital syndrome, type I; Otopalatodigital syndrome, type II; Terminal osseous dysplasia
FOXE3	1p33	601,094	AD; AR	Aortic aneurysm, familial thoracic 11, susceptibility to; Anterior segment dysgenesis 2, multiple subtypes; Cataract 34, multiple types
IFIH1	2q24.2	606,951	AD; AR	Aicardi-Goutieres syndrome 7; Immunodeficiency 95; Singleton-Merten syndrome 1
LOX	5q23.1	153,455	AD	Aortic aneurysm, familial thoracic 10
MFAP5	12p13.31	601,103	AD	Aortic aneurysm, familial thoracic 9
MYH11	16p13.11	160,745	AD; AR	Aortic aneurysm, familial thoracic 4; Megacystis-microcolon-intestinal hypoperistalsis syndrome 2; Visceral myopathy 2
MYLK	3q21.1	600,922	AD; AR	Aortic aneurysm, familial thoracic 7; Megacystis-microcolon-intestinal hypoperistalsis syndrome 1
NOTCH1	9q34.3	190,198	AD	Adams-Oliver syndrome 5; Aortic valve disease 1
PLOD1	1p36.22	153,454	AR	Ehlers-Danlos syndrome, kyphoscoliotic type, 1
PLOD3	7q22.1	603,066	AR	BCARD syndrome (lysyl hydroxylase 3 deficiency)
PRKG1	10q11.2-q21.1	176,894	AD	Aortic aneurysm, familial thoracic 8
RNASEH2A	19p13.13	606,034	AR	Aicardi-Goutieres syndrome 4
RNASEH2B	13q14.3	610,326	AR	Aicardi-Goutieres syndrome 2
RNASEH2C	11q13.1	610,330	AR	Aicardi-Goutieres syndrome 3
SAMHD1	20q11.23	612,952	AR	Aicardi-Goutieres syndrome 5
SKI	1p36.33-p36.32	164,780	AD	Shprintzen-Goldberg syndrome
SLC2A10	20q13.12	606,145	AR	Arterial tortuosity syndrome
SMAD3	15q22.33	603,109	AD	Loeys-Dietz syndrome 3
TGFB2	1q41	190,220	AD	Camurati-Engelmann disease 2; Loeys-Dietz syndrome 4
TGFB3	14q24.3	190,230	AD	Arrhythmogenic right ventricular dysplasia 1; Loeys-Dietz syndrome 5
TGFBR1	9q22.33	190,181	AD	Multiple self-healing squamous epithelioma, susceptibility to; Loeys-Dietz syndrome 1
TGFBR2	3p24.1	190,182	AD	Colorectal cancer, hereditary nonpolyposis, type 6; Esophageal cancer, somatic; Loeys-Dietz syndrome 2
TREX1	3p21.31	606,609	AD	Vasculopathy, retinal, with cerebral leukodystrophy

Phenotypes were from online Mendelian Inheritance in Man (OMIM) database

*AR* autosomal recessive; *AD* autosomal dominant

### Systematic review

We systematically searched PubMed up to December 2025 for original articles written in English. The search strategy is summarized in the Supplemental Methods. Additionally, we manually reviewed the reference lists of the included studies to identify any additional eligible articles. Case reports were not included. We also excluded studies on purely intracranial dissections, a different phenotype than CeAD. Three Authors (LC, CF and FT) independently screened studies. Conflicts during screening were resolved by the senior author (AP). Data extracted related to study details (e.g., authors, publication year), patient phenotype, screened genes, methodologic approach and results of the molecular analyses.

## Results

Overall, of the 185 patients who received the diagnosis of sCeAD during the study period, 183 (mean age, 42.0 ± 11.3 years; males, 51.3%; ischemic stroke, 73.4%; minor trauma before dissection, 25.7%) qualified for the analysis and consented to participate in this study. Clinical and demographic characteristics of the study group are summarized in Table 2. The diagnosis of CeAD was established or confirmed by MRI in all cases*.* We identified 2 (1.1%) patients carrying a CeAD-causing variant. In addition, we identified 30 (16.4%) VUS in 25 (13.6%) patients (Table S1).

**Table 2:** Demographic and clinical characteristics of the study group

	*n* (%)
Age at first stroke onset, years	42.0 ± 11.3
Sex, male	94 (51.3)
Hypertension	45 (24.5)
Diabetes	3 (1.6)
Hypercholesterolemia	19 (10.3)
Active smoking	47 (25.7)
Migraine	
Any	71 (38.7)
Migraine without aura	59 (32.2)
Migraine with aura	12 (6.5)
Dissected vessel	
Internal carotid artery	101 (55.2)
Vertebral artery	39 (21.4)
Multiple vessels*	43 (23.4)
Fibromuscular dysplasia	19 (10.4)
Presenting symptom	
Ischemic stroke	132 (72.1)
TIA	14 (7.6)
Local symptoms^†^	96 (52.4)
Retinal ischemia	0 (0.0)
Subarachnoid hemorrhage	0 (0.0)
Family history of arterial dissection^‡^	0 (0.0)

* More than one cervical vessel involved

^†^ Including Horner syndrome, cranial nerve palsies, head or neck pain, or pulsatile tinnitus

^‡^ In first- and second-degree relatives

Patient no. 1 was a 21-year-old man who was admitted because of sharp left-sided cervical pain and Horner syndrome. Medical history included surgery for right inguinal hernia at the age of 18, while physical examination revealed slightly hypermobile interphalangeal joints. Brain MRI did not show any parenchymal lesions, but dissection of the extra-cranial portion of the left internal carotid artery (ICA). The NM_000090.4:c.2959G > A:p.Gly987Ser mutation in COL3A1 in a heterozygous state was found.

Patient no. 2 was a 39-year-old woman with no history of major cardiovascular risk factors other than mild hypertension and smoking (5 cigarettes per day for 15 years before hospital admission) and an unremarkable family history, who presented with sudden onset of right-sided hemiparesis and dysarthria. Brain MRI revealed acute infarction in the territory of the left middle cerebral artery (MCA) and subcortical ischemic leukoencephalopathy along with dissection of the extra-cranial portion of the left ICA. Clinical examination was unremarkable, apart from loose skin at the sides of the neck and axillae. The patient turned out to harbor the NM_001351800.1:c.3071G > A:p.Arg1024Gln mutation in ABCC6 at the heterozygous state.

### Systematic review

Following a full-text review based on exclusion and inclusion criteria, the process resulted in a final set of 14 articles selected for data extraction and included in the systematic review [5, 9, 10, 14–24]. Overall, out of 330 patients with sCeAD, 21 (6.3%) tested positive for clinically relevant genetic variants (CRGVs). Specifically, the prevalence of these variants in each sub-group was as follows: 18 out of 77 (23.4%) patients with familial history of arterial dissection, 4 out of 33 (12.1%) patients with recurrent-multivessel arterial dissection and 1 out of 228 (0.4%) patients who had neither recurrent-multivessel events nor family history of CeAD (Table 3).

**Table 3:** Studies that have investigated candidate genes for disease-causing mutations in patients with spontaneous cervical artery dissection

Ref no.	Author, year	Gene	Chromosome	Sample size (no. of cases)	Study group	Results
[22]	Martin, 2006	Collagen, Type I, α-1 (COL1A1)	17q21.33	10	All patients with familial history of sCeAD	Negative
		Collagen, Type III, α-1 (COL3A1)	2q31	7		G157S missense mutation in 2 patients
		Collagen, Type V, α-1 (COL5A1)	9q34.2-q34.3	6		Negative
		Collagen, Type V, α-2 (COL5A2)	2q14-q32	5		Negative
[16]	Kuivaniemi, 1993	Collagen, Type III, α-1 (COL3A1)	2q31	18	6 patients had familial history of sCeAD; 2 patients had recurrent dissections	Negative
[17]	Van den Berg, 1998	Collagen, Type III, α-1 (COL3A1)	2q31	16	Unselected patients	Negative
[19]	Grond-Ginsbach, 1999	Collagen, Type V, α-1 (COL5A1)	9q34.2-q34.3	19	15 patients had ultrastructural dermal connective tissue abnormalities	Negative
[21]	Grond-Ginsbach, 2000	Elastin (ELN)	7q11.23	10	All patients had ultrastructural dermal connective tissue abnormalities	Negative
[18]	Grond-Ginsbach, 2002	Collagen, Type V, α-2 (COL5A2)	2q14-q32	10	7 patients had ultrastructural dermal connective tissue abnormalities, 2 patients had familial history of sCeAD; 1 patient had recurrent sCeAD	Negative
[15]	von Pein, 2002	Collagen, Type III, α-1 (COL3A1)	2q31	12	All patients had ultrastructural dermal connective tissue abnormalities	Negative
[14]	Morcher, 2003	ATP-binding cassette, subfamily C, member 6 (ABCC6)	16p13.1	12	All patients had ultrastructural dermal connective tissue abnormalities	Negative
[20]	Kuhlenbaumer, 2004	Collagen, Type VIII, α-1 (COL8A1)	3q12.3	13	1 patient had with familial history of sCeAD	Negative
		Collagen, Type VIII, α-2 (COL8A2)	1p34.2	13		Negative
[23]	Pezzini, 2011	Transforming growth factor-beta receptor, type 1 (TGFBR1)	9q22.33	56	1 patient had recurrent sCeAD	
		Transforming growth factor-beta receptor, type 2 (TGFBR2)	3p24.1	56	1 patient had recurrent sCeAD	TGFBR2 c.412 T > C p.C138R in 1 patient TBFBR2 c.1115 A > G p.K372R in 1 patient
[12]	Giossi, 2014	Transforming growth factor-beta receptor, type 1 (TGFBR1)	9q22.33	84	unselected	Negative
		Transforming growth factor-beta receptor, type 2 (TGFBR2)	3p24.1	84	unselected	Negative
		SMAD family member 3 (SMAD3)	15q22.33	62	unselected	Negative
		Transforming growth factor-beta 2 (TGFB2)	1q41	62	unselected	Negative
		Collagen, Type III, α-1 (COL3A1)	2q31	10	unselected	Negative
		Solute carrier family 2 (facilitated glucose transporter), member 10 (SLC2A10)	20q13.12	3	unselected	Negative
		Fibrillin 1 (FBN1)	15q21.1	2	unselected	Negative
		Actin, alpha 2, smooth muscle, aorta (ACTA2)	10q23.31	2	unselected	Negative
[9]	Grond-Ginsbach, 2017	Targeted NGS (11 genes)*		18	All patients with familial history of sCeAD	
[10]	Traenka, 2019	Targeted NGS (30 genes)†		43	25 patients had familial history of sCeAD; 17 patients had recurrent dissections	FBN1 Arg244Trp in 1 patient COL3A1 Gly214Ser in 1 patient COL4A1 Pro116Leu in 1 patient COL3A3 Leu1474Pro and COL3A4 Gly972Arg in 1 patient COL5A1 Arg65Trp and Val 172Phe in 1 patient
[14]	Erhart, 2022	Whole exome sequencing (WES) and and copy number variation (CNV) analysis		4	All patients had sCeAD and aortic dissection; 2 patients had familial history of sCeAD; 3 patients had ultrastructural dermal connective tissue abnormalities	COL5A2 Gln856Glu in 1 patient in WES; dup(16)(p13.11) NC_000012.11:g.14916662_16306102dup in 1 patient; del(2)(q32.2)(189401614–190055557) x 1 in 1 patients

* genes included in the panel: ACTA2, MYH11, FBN1, COL3A1, COL4A1, TGFBR1, TGFBR2, TGFB2, SMAD3, MYLK, SLC2A10

^†^ genes included in the panel: ACTA2, COL3A1, COL4A1, COL4A3, COL4A4, COL4A5, COL5A1, COL5A2, EFAMP2, ELN, FBN1, FBLN5, FLNA, FOXE3, LOX, MFAP5, MYH11, MYLK, NOTCH1, PLOD1, PLOD3, PRKG1, SKI, SLC2A10, SMAD3, SMAD4, TGFB2, TGFB3, TGFBR1, TGFBR2

## Discussion

The analysis of the largest series of consecutive subjects with sCeAD ever investigated by a targeted NGS approach showed that, at least in unselected patients, the pre-test probability of identifying CRGVs is extremely low, being approximately 1%. This finding is in line with the sparse data of the literature mainly derived from preliminary analyses, carried out by first-generation sequencing approach, on small cohorts and a limited number of screened genes, in which the prevalence of CGRVs carriers was approximately 0.4%. Furthermore, since about 1 in 4 patients in those studies had undergone molecular analysis based on the detection of ultrastructural dermal connective tissue abnormalities, it is reasonable to assume that such percentage is even lower and the contribution of rare genetic variants in the pathogenesis of sporadic CeAD is, therefore, negligible. Conversely, the systematic review of the available literature indicates that the pre-test probability is considerably higher in subgroups of patients selected based on clinical-radiological phenotype (involvement of multiple vessels, particularly in extra-cervical districts, in addition to CeAD) and, most of all, family history of arterial dissection, in which it is close to 24%.

A common clinical issue for practicing neurologists is the selection of subjects to be screened for monogenic disorders because of the potential in identifying CRGVs carriers. However, how to select sCeAD patients for specific genetic testing remains a challenge. The results of our analysis clearly indicate that carrying out systematic molecular screening – even using advanced NGS techniques – on all patients with sCeAD who do not show obvious clinical features of HCTD is unwarranted. In the absence of further clinical or biological markers, searching for CRGVs should be restricted, for the time being, to cases with proven familial history of arterial dissection or involvement of multiple vessels in different arterial districts. The clinical impact of these findings is limited by the fact that familial occurrence of sCeAD is exceptional [25], even though CeAD might be an underdiagnosed condition [26] and that involvement of extra-cervical districts is not easily detected by routine diagnostic approaches, though it accounts for up to 14.8% of sCeAD cases according to the results of a recent study [27]. Obviously, it cannot be ruled out, in principle, that our panel of 38 candidate genes is too selective and other CRGVs might have been identified had the panel comprised further genes, which would make the rate of positive findings higher. However, this seems rather unlikely, since our panel includes all the genes linked to known HCTD.

The scenario that emerges from the results summarized above differs significantly from that seen in other non-atherosclerotic vasculopathies supposed to have pathogenetic mechanisms in common with sCeAD. This is the case, for example, of TAAD, for which it is estimated that almost 25% of patients have pathogenic mutations in genes coding for components of the ECM (e.g., COL3A1, FBN1, LOX), smooth muscle cell function (e.g., ACTA2, MYH11, MYLK, PRKG1) or the transforming growth factor-β signaling pathway (e.g., SMAD3, TGFB2, TGFBR1, TGFBR2) [28]. This has led the European Society for Vascular and Endovascular Surgery to recommend genetic testing in TAAD patients younger than 40 years [29] and some Authors to extend the screening to patients younger than 55 years even in the absence of known familial predisposition [30]. The flip side of the coin, which should not be overlooked, is that, although the detection of CGRVs among patients with sporadic sCeAD is uncommon, carriers identified in our series or in many previous ones did not exhibit clinical or radiological features suggestive of HCTD. These cases would, therefore, have remained undiagnosed had systematic molecular screening not been carried out on all sCeAD patients, with obvious implications in terms of clinical management and family counselling.

From a biological point of view, the results of our analysis support the hypothesis that a connective tissue disorder predisposes to arterial wall weakness in sCeAD patients. In particular, perturbations of the ECM, which accounts for up to 60% of the dry weight of a large vessel [31], are supposed to have a key pathogenic role. ECM is critical to the structure and function of the arterial wall as it ensures elasticity and distensibility and plays an essential role in signal transmission, both directly, by interacting with adhesion molecules such as integrins, and indirectly, as a reservoir for signaling factors. Differences in ECM composition provides the specific mechanical and biochemical properties of the vessel wall and for maintenance of arterial homeostasis [32]. There are two major macromolecules within the arterial ECM: elastic fibers and collagen fibers. Elastic fibers comprise a diverse range of ECM species, of which elastin is the major component. Decrease in elastin expression results in thickening of lamellar units and increase in vascular smooth muscle cells (VSMCs), which is thought to compensate for the reduced elasticity normally provided by the elastin and has been linked to hypertension in both mice and humans [33]. Collagen fibers, which consist of bundles of fibrils that are formed from triple-helix chains, provide different mechanical properties, such as stiffness or elasticity, which vary according to the type of collagen [34]. In the arterial wall, collagen is the main factor driving the contractile changes that occur and is believed to be responsible for the stiffness of vessels [35]. Based on these pathophysiological considerations, it is not surprising that pathogenic variants of COL3A1, causing defect of type III collagen and leading to the fragility of arterial wall and ABCC6, causing elastic fibers fragmentation and abnormalities, may cause sCeAD, even though neither of our two patients presented the full phenotypic spectrum of the corresponding disease entity (vascular Ehlers-Danlos syndrome [vEDS] for COL3A1 and Pseudoxanthoma Elasticum [PXE] for ABCC6) and conclusive clinical signs were lacking in both cases.

In conclusion, the results of our study and the systematic review of the literature indicate that the prevalence of CGRVs in genes associated with connective tissue disorders or vascular anomalies is extremely low in patients with CseAD who have not been selected a priori and their impact on the epidemiology of the disease is, therefore, negligible. Conversely, the detection of dissections involving multiple arterial districts other than the cervical-cephalic region, and, even more remarkable, a family history of arterial dissection, is strongly predictive of disease-causing mutations. A targeted sCeAD-specific high-throughput sequencing panel, allowing the simultaneous testing of multiple genes underlying a single disease phenotype, might be regarded as a cost-efficient approach in cases of sCeAD suspected to be genetically determined.

## Supplementary Information

Below is the link to the electronic supplementary material.

Supplementary Material 1
