# Supplementary material for: Hereditary connective tissue disorders in unselected patients with spontaneous cervical artery dissection: a targeted next generation sequencing approach and systematic review
Source: Neurol Sci. 2026 Sep 2;47(9):753. doi: 10.1007/s10072-026-09308-6 (PMC13538227; doi:10.1007/s10072-026-09308-6)
Supplement: Supplementary file 1 [file 10072_2026_9308_MOESM1_ESM.docx]

**Supplementary Material**

**Supplemental Methods: search strategy**

To explore the relationship between known HCTD and sCeAD, we combined (“carotid artery, internal, dissection”[Mesh] or “vertebral artery dissection”[Mesh] or “aneurysm, dissecting”[Mesh] or “cervical artery dissection$” or ((“carotid artery, internal”[Mesh] or “carotid arteries”[Mesh] or “vertebral artery”[Mesh]) and “dissection$”)) with the following terms: (“ehlers-danlos syndrome”[Mesh] or “marfan syndrome”[Mesh] or “cutis laxa”[Mesh] or “pseudoxanthoma elasticum”[Mesh] or “loeys-dietz”[Mesh] or “polycystic kidney, autosomal dominant”[Mesh] or “arterial tortuosity syndrome”[Mesh]). This search yielded 73 citations, from which the relevant studies were selected.

**Table S1**

**Summary of variants of unknown significance (VUS) detected in the study group.**

^†^ in 2 patients

**Table S1**
